# Measuring the Conservation Value of Tropical Primary Forests: The Effect of Occasional Species on Estimates of Biodiversity Uniqueness

**DOI:** 10.1371/journal.pone.0009609

**Published:** 2010-03-09

**Authors:** Jos Barlow, Toby A. Gardner, Julio Louzada, Carlos A. Peres

**Affiliations:** 1 Lancaster Environment Centre, Lancaster University, Lancaster, United Kingdom; 2 Departamento de Biologia, Universidade Federal de Lavras, Lavras, Minas Gerais, Brazil; 3 Department of Zoology, University of Cambridge, Cambridge, United Kingdom; 4 School of Environmental Science, University of East Anglia, Norwich, United Kingdom; Centre National de la Recherche Scientifique, France

## Abstract

**Background:**

Developing effective conservation plans for multi-functional landscapes requires an accurate knowledge of the relative conservation value of different land-uses. A growing number of tropical ecologists have evaluated conservation value using the number (or proportion) of species that are unique to primary or old-growth forests. However, estimates of the conservation value of modified land-uses may be inflated by the presence of occasional species (e.g. singletons and doubletons) that may be unable to exist as viable populations in isolation.

**Methodology/Principal Findings:**

We use a unique 15-taxa dataset from a mixed-use forest landscape in the Brazilian Amazon to test the hypothesis that the removal of occasional species from sample data can increase estimates of the value of primary forest for biodiversity conservation.

**Conclusions/Significance:**

Estimates of conservation value that are based on the proportion of species that are unique to tropical primary or old-growth forests are highly sensitive to decisions researchers make regarding the inclusion or exclusion of occasional species. By removing singletons from modified forest samples, and considering only those species known to occur in primary forest, we almost double estimates of the conservation value of tropical primary forests.

## Introduction

Making informed decisions on the design of multi-functional landscapes for biodiversity conservation requires an accurate knowledge of the relative conservation value of the different land-uses [1]. The ability to accurately determine the relative conservation values of undisturbed primary and human-modified environments is of particular importance. However, quantifying these values can be extremely difficult in complex species-rich ecosystems. This problem is particularly acute in the humid tropics where most species are locally rare, many remain undescribed, and there is a lack of ecological or biological information available for those species that are known to science [2], [3], [4].

Given the critical lack of detailed information on species ranges, preferred habitats and functional roles, comparisons of the biodiversity value of different land-uses invariably depend upon relatively simple metrics. In recent years, a growing number of tropical ecologists have evaluated conservation value using the number (or proportion) of species that are unique to primary or old-growth forests when compared to other land-uses within the wider landscape [5], [6], [7], [8]. Despite its simplicity, this method has many advantages as it facilitates comparisons between studies [6], [9], and is intuitive and easier for non-scientists to understand than many alternative measures of conservation value like similarity indices (see Materials and Methods).

However, it is possible that this metric is highly sensitive to occasional species in samples. There are good reasons why species may be naturally rare [10], but in many cases this rarity may be a sampling artifact. For example, some of the individuals recorded in biodiversity samples could represent transient or sink populations and spill-over effects could mean that occasional species are recorded in modified land-uses even though they are unable to sustain viable populations in the long-term when isolated from source populations in primary habitat. This could lead scientists to underestimate the conservation value of primary forests compared to alternative land-uses [5], [11], [12]. These occasional or rare species are thought to dominate species sample data from systems around the world [10], and their presence in samples could distort indices of uniqueness.

In an ideal world, we would have direct assessments of the viability of all species in biodiversity samples, and could use this to inform our estimates of conservation value of different land-uses. However, we are highly unlikely to ever fulfill this for the vast majority of tropical forest species, and are thereby forced to use species sample-abundances as a proximate estimate of its viability in a given environment (while acknowledging the abundance does not always provide a good proxy of habitat quality; [13]). This presents a dilemma regarding occasional species, as all else being equal we have much greater confidence about the viability of species that are abundant that species that are rare in samples. One common approach used to address this problem is to simply remove occasional species from analyses [14], so inferences are limited to those species that we have most confidence in. This is equivalent to discarding species that were only recorded using a particular sampling technique that is known to be unreliable. However, in this case there is a risk of losing valuable information regarding patterns of conservation value. Furthermore, determining which of these occasional species are genuinely rare (i.e. having either a small geographic range, narrow habitat breadth or a low local density; [15] is difficult and highly context-dependent [16] and requires detailed biological information that remains unavailable for the vast majority of species [17], and especially for those found in tropical forests.

An alternative method is to remove occasional or rare species according to their rank abundance or occupancy [10]. However, rules regarding how many species to remove, or where to remove them from, are very subjective. A search of the literature quickly reveals a bewildering array of criteria for classifying or excluding rare and occasional species from ecological analyses, including removing species with <3 occurrences [18]; species that are not present in at least 5% of the samples [19]; species representing ≤1% of any sample [20]; and species that fail to represent at least 2% (in term of abundance and biomass) of every monthly sample from each sampling station [21].

Given the frequency with which these removal rules have been applied, it is alarming that so little is known about how these essentially arbitrary decisions affect estimates of conservation value. This question has particular applied relevance to the current tropical conservation research agenda as it has recently been argued that agricultural abandonment and subsequent recovery of secondary forests on degraded land could help offset the biodiversity lost from the clearance of old-growth tropical forests [22]. Understanding the extent to which secondary forests and modified forest land-uses such as plantation forestry may provide a “safety net” for tropical forest species depends on having a robust understanding of their conservation value [2], [6], [23].

We use a comprehensive dataset of 15 taxa sampled in primary, secondary and plantation forests in the northeastern Brazilian Amazon to explore how occasional species affect estimates of conservation value. By sequentially removing different abundance classes of occasional species, we test the hypothesis that occasional species can significantly decrease estimates of the conservation value of primary forests (or, conversely, increase the estimated conservation value of modified land-uses). The rationale for this is based on the possibility that spill-over effects could lead to the detection of a disproportionately high number of occasional species in modified land-uses, although the lack of biological data on almost all tropical forest species means we are unable to examine the mechanistic causes behind the observed patterns. We use two different removal rules (removing occasional species from all forest types simultaneously, or removing occasional species from modified land-uses only) and two alternative metrics of uniqueness (the proportion of the entire species pool unique to primary forest, and the proportion of species that were recorded in primary forest that were also unique to primary forest). Although most tropical forest biologists are aware that the removal of the rarest species from analyses will inevitably affect estimates of conservation value based on unique species, this is the first time the sensitivity of these commonly used conservation value metrics has been assessed in any systematic way.

## Materials and Methods

### Data Collection

Field data were collected within the Jari region of Pará, north-eastern Brazilian Amazonia. Sampling protocols, vegetation descriptions, and landscape structure are described in detail elsewhere [5], [11], [24]. Data on 14 faunal taxa and one plant taxon (trees and lianas) were collected from 15 study sites, including five primary forests, five 14–19 year old secondary forests, and five 4–6 year old *Eucalyptus* plantations. These forest blocks were spatially independent (mean distance between primary, secondary and Eucalyptus sites was 30 km (range = 14–67 km), 9 km (range = 4–44 km) and 11 km (range = 7–50 km), respectively), and the potential influence of adjacent land-uses was minimized as forest blocks were very large in comparison with most previous studies (mean size of Eucalyptus and secondary forest blocks was 1687 ha (range = 574–3910 ha) and 2682 ha (range = 1079–3508 ha), respectively). However, the modified land-uses were embedded in a vast area of relatively intact primary forest, and it seems likely that the secondary and plantation forest samples could have recorded many occasional species that would not exist without the favourable landscape context [11], [25].

A large multi-national research team spent over two years and >18,200 person-hours sampling and identifying the focal taxa, recording a total of 1441 species. Taxa (no. of records; no. of species) were grouped following [5], and included leaf-litter amphibians (1,739; 23), lizards (1,937; 30), large mammals (1,227; 30), small non-volant mammals (219; 32), bats (4,125; 54), birds (6,865; 255), epigeic arachnids (3,176; 116), scavenger flies (5,365; 30), dung beetles (9,203; 85), fruit-feeding butterflies (10,987; 128), fruit flies (5,085; 38), moths from the Sphingidae, Saturnidae, and Arctiidae (1,848, 335), grasshoppers (942; 44) and orchid bees (2,363; 22), and tree and woody liana *genera* (8,077; 219). Almost all species were native to South America (with some obvious exceptions such as *Eucalyptus*).

### Defining Conservation Value and Occasional Species

We define the conservation value of old-growth forest by the percentage of species that do not occur in the surrounding modified land-uses: put simply, the more species that are unique to old-growth forests, the greater their importance for conservation. We recognize that this definition represents a gross simplification as rare, declining, phylogenetically distinct, and functionally important species are generally perceived as having a higher conservation value [26]. However, the lack of baseline information on the overwhelming majority of species means we weight all species equally.

Although similarity indices are becoming increasingly sophisticated, and can be used as alternative measures of conservation value [27], we do not address them here as the robustness of these indices to sampling representation and rare species has already been addressed in detail before [28], [29]. Furthermore, while some of these similarity indices are robust to incomplete sampling and low sample effort, these require a level of data complexity that is not often available in meta-analyses of multi-taxa datasets (for example, estimation of probabilities of detection requires that the community is sampled several times and is divided into multiple spatially explicit subsamples). Finally, similarity indices are much less intuitive for non-scientists than a percentage of species unique to a forest type.

Species encountered in modified land-uses could have few occurrences either because they are naturally rare in samples [17], or because individuals could be reproductively immature or juvenile individuals [30], vagrants [10], or could represent long-term sink populations [31]. However, we lack the baseline information or long-term monitoring required to distinguish frequent from occasional species [17], [32]. Instead, we define occasional species as those that form the tail of the species abundance distribution in each forest type, with 10 or fewer records (on average, a species with 10 captures in a forest type would only have been recorded twice at each site). In practice, this is the only objective way of defining occasional species in relatively short-term studies that compare the conservation value of different land-uses in tropical forest regions (i.e. the vast majority of field research to date).

### Data Analysis

We grouped data from replicate sites within each land-use type. We examine how estimates of the conservation value of primary forest vary following the sequential removal of different abundance classes, defined as species that were recorded once, twice and three times, etc, in each forest type. We use two different metrics to define the conservation value of primary forest in our samples: The proportion of the entire species pool that is unique to primary forest in our samples, and the proportion of primary forest species that are unique to primary forest (herafter, primary forest species are defined as those that were sampled at least once in any of the primary forest sites). We applied two different removal rules, removing occasional species from all land-uses simultaneously, or only removing species from modified land-uses. The combination of these removal rules and uniqueness metrics provides four different estimates of the conservation value of primary forest:

1. The proportion of all species that was unique to primary forest, simultaneously removing occasional species from all land-uses.
2. The proportion of primary forest species that was unique to primary forest, simultaneously removing occasional species from all land-uses.
3. The proportion of all species that was unique to primary forest, simultaneously removing occasional species from modified land-uses only.
4. The proportion of primary forest species that was unique to primary forest, simultaneously removing occasional species from modified land-uses only.

Because the sequential removal of occasional species excludes a different proportion of the overall abundance of each taxon, we also calculate the proportion of the total abundance and species richness that were depleted for each taxon.

## Results

**Figure 1:**
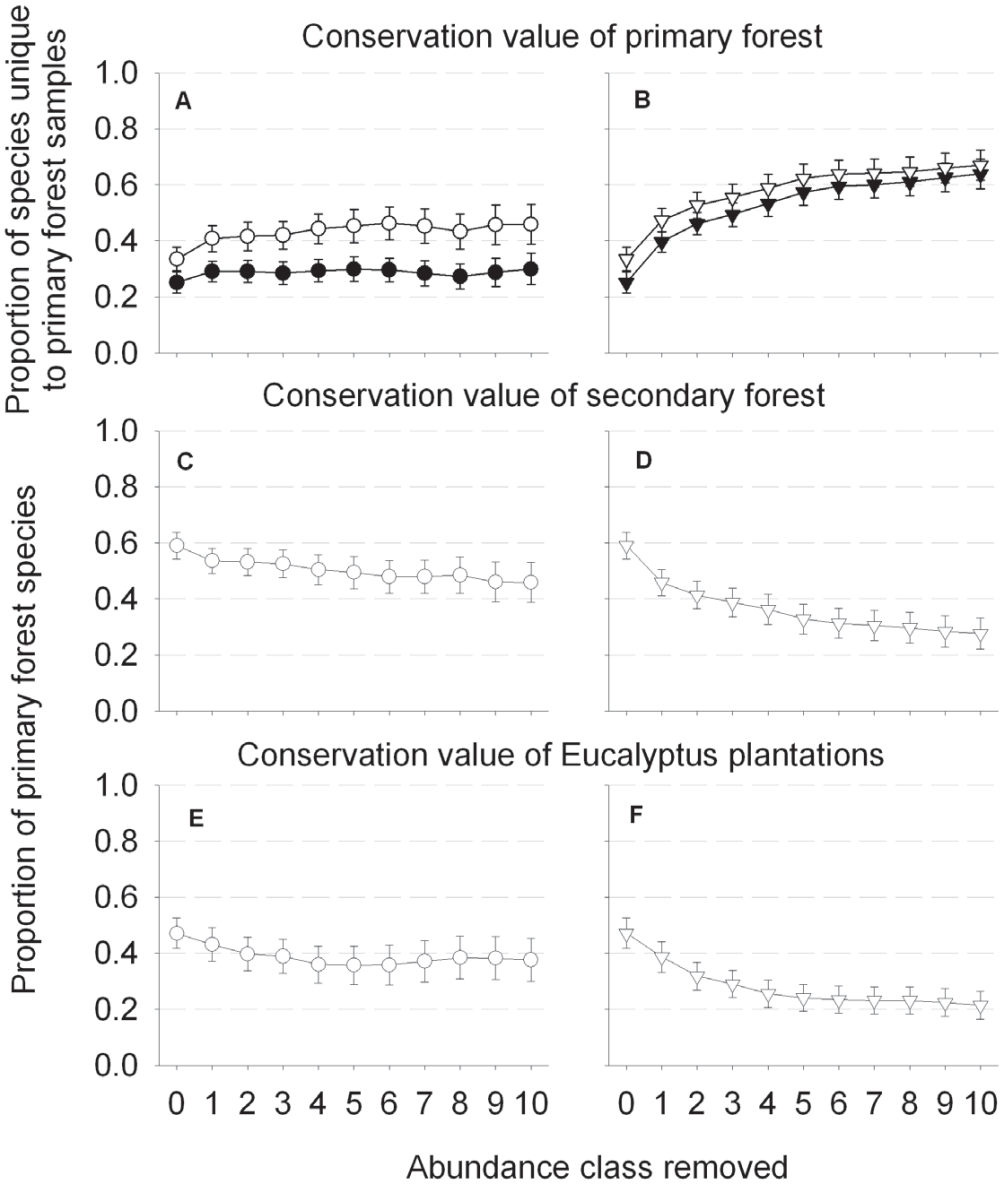
The conservation value of forests in the Jari landscape. Panels show how the metric of conservation value changes in primary forest (a & b), secondary forest (c & d) and Eucalyptus plantations (e & f) as an increasing number of abundance classes of occasional species are sequentially removed from the analysis. Values represent the mean proportion (± SE) from 15 vertebrate, invertebrate and plant taxa sampled. Panels on the left (a, c & e) represent values when occasional species are removed from all forest types simultaneously (symbols as circles), while panels on the right (b, d, & f) represent values when occasional species are only removed from modified forest types (symbols as triangles). Solid and open symbols (panels a & b) represent the proportion of the entire species pool, and the proportion of primary forest species (i.e. only species that were recorded at least once in primary forest), respectively.

**Figure 2:**
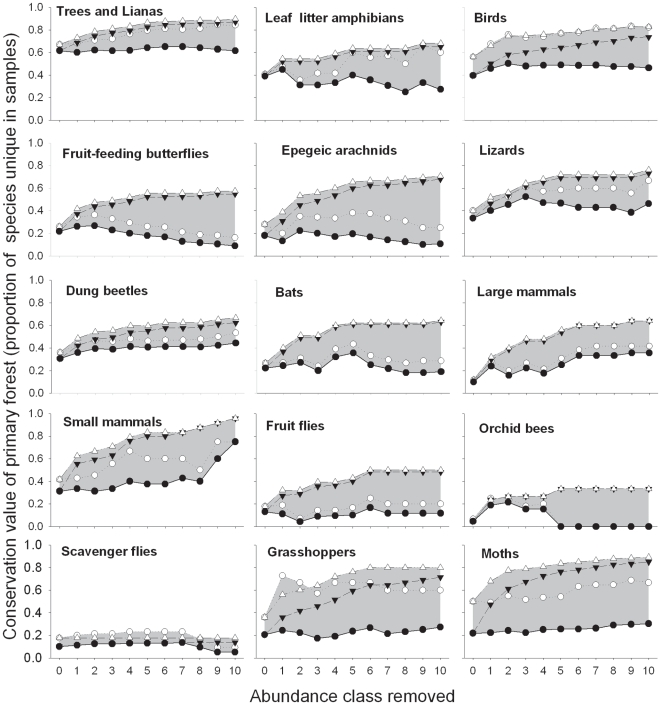
The conservation value of primary forest (measured as the proportion of species unique to primary forest samples) for 15 vertebrate, invertebrate and plant taxa, following the sequential removal of abundance classes. Lines within each panel show different selection criteria and removal rules, and the grey shaded area indicates the region between the upper and lower estimates of uniqueness. Circles represent values when occasional species are removed from all forest types simultaneously, and triangles represent the removal of occasional species from modified forest types only. Solid and open symbols represent the proportion of the entire species pool, and the proportion of primary forest species (i.e. only species that were recorded in primary forest), respectively. Order of panels follows Barlow *et al*. (2007).

Overall, the removal of occasional species consistently increased estimates of the conservation value of primary forest (Figure 1a and 1b) while reducing estimates of the conservation value of both secondary forests and *Eucalyptus* plantations (Figure 1c–f). Irrespective of the removal rule or uniqueness metric, there was a fundamental difference among different taxa in the proportion of species considered to be unique to primary forest (Figure 2, and see [5]). The consequences of removing occasional species were also highly variable between taxa. Arachnids, fruit-flies and fruit-feeding butterflies were either strongly affected by the removal rule, the uniqueness metric, or both, while other taxa were less sensitive (Figure 2)

The removal of singletons had a greater effect on conservation value estimates than the sequential removal of doubletons, trebletons, etc (Figures 1 and 2). The removal of additional occasional species (those recorded more than three times) had little or no overall effect on the most unbiased uniqueness metrics which excluded occasional species from both primary and modified land-uses, regardless of whether we considered the proportion of all primary forest species or the proportion of the entire species pool (Figure 1a). Unsurprisingly, when occasional species were only removed from modified land-uses, a larger proportion of species were considered to be unique to primary forests (Figure 1b), but this rise approached an asymptote after the removal of species recorded five times or less.

### Sample Representation

**Figure 3:**
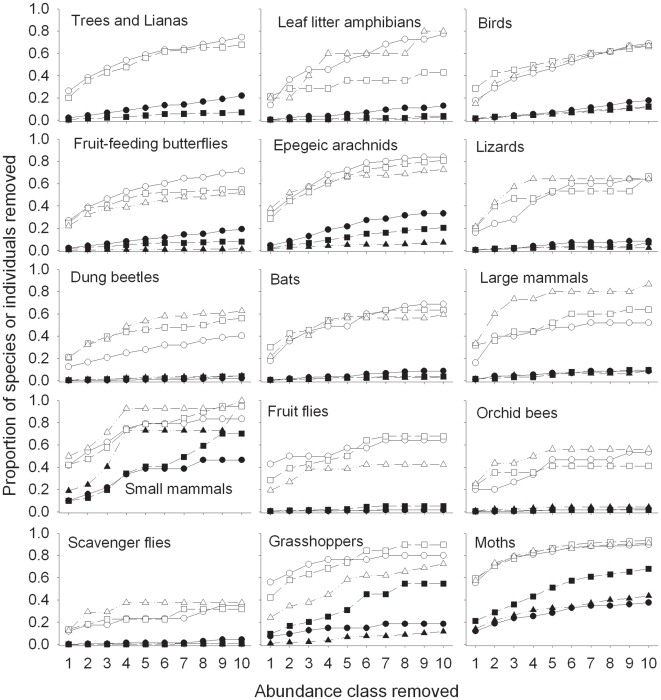
Patterns of rarity in the species data used in this paper. Panels show the cumulative proportion of species (black symbols) and individuals (open symbols) removed from primary forest (circles), secondary forests (squares) and tree plantations (triangles) (i.e. following the removal of singletons, doubletons, and so on, up to those species that were recorded ≤10 times in each forest type).

Occasional species accounted for a highly variable proportion of the species richness and total abundance of the different taxa (Figure 3). Overall, singletons accounted for 3% of the total abundance and 28% of the species from the forest treatments, while doubletons accounted for 2% and 12%, and trebletons 2% and 6%, respectively. There was substantial variation between taxa and between forest types: singletons alone accounted for between 55–60% of all species in the three forest types in species rich taxa like moths, but just 12–14% of the species of scavenger flies (Figure 3).

Sample representation was estimated by dividing observed richness in each forest type by the average of three abundance-based richness estimators (Chao 1, Jack 1 and ACE; see Colwell, 2005). Overall, estimated sample representation was generally high across taxa (>70% for 11, 12, and 13 of the 15 taxa sampled in primary, secondary and plantations, respectively; see [24]). The mean sample representation for all forest types was not correlated with the proportion of species in each taxa that were considered unique to primary forest (r = −0.02, n = 15, P = 0.9). Within treatment sample representation can also be assessed visually from species accumulation curves shown in previous manuscripts using the same data (see [5]).

## Discussion

The development of effective conservation management plans that encompass entire landscapes requires a detailed understanding of the biodiversity consequences of land-use change and landscape modification [33], [34]. Within the humid tropics, this understanding is severely limited by our poor knowledge of species biology, making it almost impossible to determine whether occasional species recorded in modified land-uses represent viable populations.

As a result, the biodiversity uniqueness of undisturbed primary forests is shrouded by many layers of uncertainty. Estimates are particularly sensitive to decisions researchers make regarding the inclusion or exclusion of occasional species, or how many to exclude (Figures 1 and 2); whether occasional species are excluded from all land-uses, or only modified land-uses (Figure 1, comparing panels a, c and e with b, d and f); whether the metric of conservation value considers the entire species pool for that landscape, or only those species known to exists within the land-use of conservation interest (e.g. primary tropical forests in our case) (Figures 1a & b); and the choice of study taxa (Figure 2). Moreover, estimates of uniqueness are also likely to be affected by the level of taxonomic resolution achieved by researchers, particularly when apparently abundant genera are composed of many rare species (as is likely to be the case for many genera of trees and lianas). If primary forests hold more rare species then anthropogenic land-uses, then a low taxonomic resolution could also lead researcher to underestimate the unique conservation value of primary forests.

Our results show that the lowest proportion of species are considered to be unique to the land-use of interest if no occasional species are removed and the proportion is calculated based on the entire species pool [5]. By contrast, estimates of uniqueness are much higher if an increasing number of occasional species are removed (from either pool), if occasional species are only removed from modified land-uses, and if the proportion is based on the pool of species that are known to inhabit primary forest.

By presenting the uncertainty inherent in almost all biodiversity sample data, we provide a more transparent estimate of the importance of areas of primary forest in multiple-use landscape mosaics. Yet the metrics that make up this upper and lower bound should not be weighted equally. The lower bound of uniqueness will almost certainly underestimate the biodiversity uniqueness of primary forests, because the metric includes many species that are not found in undisturbed land-uses (i.e. open-area and generalist species), and because the removal rule removes many of the rare species that are so characteristic of tropical primary forests.

In our opinion, the upper bound is much more likely to provide an accurate estimate of the conservation value of primary forests. This is because there is much stronger theoretical support for 1) removing occasional species from modified forest sites only (removing species from primary environments is likely to remove viable populations of threatened or rare species) and 2) only valuing species that were recorded at least once in primary forest (it makes little sense to include species that are recorded outside of primary forest but never within it, as these are likely to be species of lowest conservation concern). Although this metric could overestimate conservation value, as some of the occasional species recorded outside primary forest inevitably represent viable populations, we believe it is the most useful metric to use under a precautionary approach, especially when considered against the full range of uncertainty. Finally, it is important to note that we did not remove occasional species from the primary forest only, while leaving them in the modified land-uses. We chose not adopt this approach as we can see no rational biological justification for assuming that occasional species would be able to maintain viable populations in modified land-uses, but would not be able to so in native primary forests.

### How Irreplaceable Are the Primary Forests of Jari

Without removing any occasional species, 25% of the entire species pool (averaging across all 15 taxa) was unique to primary forest when compared to alternative land-uses, here represented by young (14–19 year old) secondary forests and 4–6 year old *Eucalyptus* plantations [5]. However, this figure rose to 34% if we only consider species that were recorded at least once in primary forest sites, which targets those species of particular conservation value because they are by definition more sensitive to forest loss than species able to inhabit anthropogenic environments. The removal of singletons from all forest types increased the average estimate of biodiversity uniqueness to 41%, and the removal of singletons from land-uses other than primary forests increased the estimate further still, to 47%. In short, we almost doubled the estimate of the conservation value of primary forest by excluding species that were only recorded once outside primary forest, and by limiting the comparison to species known to occur in primary forest. In doing so we also reduced our estimates of the value of the modified forests in the Jari region of Amazonia. The secondary forests and *Eucalyptus* plantations held 59% and 47% of all primary forest species, respectively, without the removal of occasional species [5], yet these values fell to 46% and 39% if singletons were excluded from these samples, and were further reduced by the exclusion of more occasional species.

### Variation between Taxa

It is also important to note that estimates of conservation value were much higher for some taxa than others, and some taxa were more sensitive to the exclusion of occasional species (Figure 2). The exclusion of singletons increased the percentage of bird species (range 46–68%) and tree and liana genera (60–73%) that would be expected to be lost if primary forests were converted to landscapes dominated exclusively by secondary forests and tree plantations. These values are considerably higher than those reported previously based on the most conservative measure of biodiversity uniqueness (40% for birds and 62% for tree and liana genera [5]), and provide strong justification for a focus of conservation efforts on the protection of remaining areas of primary forests, wherever this is possible.

### Should Biodiversity Sampling Ignore Rare Species

Occasional and rare species are often difficult to sample and identify, and where it is attempted this work occupies a disproportionate amount of research time [35]. Removing these rare species from the identification process and subsequent analyses may be cost effective, helping to maximise statistical power (e.g. by increasing the number of sites that are sampled) for relatively little loss of ecological information [36]. Although our results demonstrate the importance of considering occasional species from some analyses, we do not advocate ignoring them in the identification process. First, it is obviously impossible to know which species are rare without attempting to identify all specimens that are collected. Second, there are additional benefits to identifying rare species, especially in the fields on taxonomy and biogeography. Third, rare species are often the most vulnerable to disturbance or land-use change [37]. Finally, without identifying rare species, it would not be possible to understand the sensitivity of patterns of conservation value to differences in data analysis.

### Conclusions

Although it seems obvious to ecologists that relatively intact or pristine environments have a high conservation value, this does not always translate through to environmental policy [38]. It is therefore vital that conservation biologists are able to make accurate assessments of the biodiversity consequences of land-use change. Inferences researchers make about the distribution and abundance of species in relation to their local environment are unavoidably influenced by a multitude of decisions concerning research design, execution and interpretation [12]. We demonstrate that estimates of the conservation value of primary forest relative to human-modified land-uses are highly sensitive to decisions regarding occasional species when based on the proportion of unique species. We suggest that scientists using this metric should consider the influence of occasional species on their results, and should report upper and lower bounds on estimates rather than the exact numbers which are almost certainly incorrect.
